# Preoperative Stereotactic Radiosurgery for Brain Metastases

**DOI:** 10.3389/fneur.2018.00959

**Published:** 2018-11-13

**Authors:** David M. Routman, Elizabeth Yan, Sujay Vora, Jennifer Peterson, Anita Mahajan, Kaisorn L. Chaichana, Nadia Laack, Paul D. Brown, Ian F. Parney, Terry C. Burns, Daniel M. Trifiletti

**Affiliations:** ^1^Department of Radiation Oncology, Mayo Clinic, Rochester, MN, United States; ^2^Department of Radiation Oncology, Mayo Clinic, Phoenix, AZ, United States; ^3^Department of Radiation Oncology, Mayo Clinic, Jacksonville, FL, United States; ^4^Department of Neurological Surgery, Mayo Clinic, Jacksonville, FL, United States; ^5^Department of Neurological Surgery, Mayo Clinic, Rochester, MN, United States

**Keywords:** preoperative, neoadjuvant, stereotactic radiosurgery (SRS), postoperative, brain metastases, local recurrence, radionecrosis, leptomeningeal disease

## Abstract

Stereotactic radiosurgery (SRS) is increasingly utilized to treat the resection cavity following resection of brain metastases and recent randomized trials have confirmed postoperative SRS as a standard of care. Postoperative SRS for resected brain metastases improves local control compared to observation, while also preserving neurocognitive function in comparison to whole brain radiation therapy (WBRT). However, even with surgery and SRS, rates of local recurrence at 1 year may be as high as 40%, especially for larger cavities, and there is also a known risk of leptomeningeal disease after surgery. Additional treatment strategies are needed to improve control while maintaining or decreasing the toxicity profile associated with treatment. Preoperative SRS is discussed here as one such approach. Preoperative SRS allows for contouring of an intact metastasis, as opposed to an irregularly shaped surgical cavity in the post-op setting. Delivering SRS prior to surgery may also allow for a “sterilizing” effect, with the potential to increase tumor control by decreasing intra-operative seeding of viable tumor cells beyond the treated cavity, and decreasing risk of leptomeningeal disease. Because there is no need to treat brain surrounding tumor in the preoperative setting, and since the majority of the high dose volume can then be resected at surgery, the rate of symptomatic radiation necrosis may also be reduced with preoperative SRS. In this mini review, we explore the potential benefits and risks of preoperative vs. postoperative SRS for brain metastases as well as the existing literature to date, including published outcomes with preoperative SRS.

## Introduction

The incidence of brain metastases (BrM) is increasing with approximately 175,000–200,000 patients developing BrM in the United States yearly (1, 2). This increase is likely multifactorial and related to both increased detection and an increase in actual development of BrM in cancer patients. Patients are now more frequently surveilled with dedicated imaging, leading to greater rates of detection. And, improvements in local and systemic options for cancer patients are improving overall survival (OS), allowing more time for BrM to occur, especially in the setting of targeted agents that may not penetrate the central nervous system (CNS). Indeed, up to 20–30% of patients with solid tumors may ultimately develop BrM (3, 4). The rate of BrM can be even higher in select populations, such as patients with HER2 positive breast cancer receiving directed therapy (5). Additionally, patients with BrM are living longer. For example, ALK-rearranged lung cancer patients may live a median of 49.5 months *afte*r the development of BrM (6). Thus, optimal management becomes an important consideration, including balancing the effectiveness of treatment with associated toxicity. Here, we present a review of the management of surgically resected BrM with consideration for strategies to improve local tumor control and potentially decrease toxicity in long term survivors, focusing on preoperative stereotactic radiosurgery (SRS) as one such novel strategy.

## Surgery for brain metastases

Surgery continues to play a significant role in the management of BrM for decompression and relief of symptoms secondary to mass effect, tissue diagnosis including relevant molecular analysis, local control in select cases such as larger lesions, and/or for a combination of these reasons (7). Surgery has been associated with improved overall survival (OS), especially in the setting of a single or solitary BrM (8, 9). In a seminal study by Patchell et al. patients randomized to surgery followed by adjuvant whole brain radiation therapy (WBRT) lived longer as compared to those receiving WBRT alone, with a median overall survival (OS) of 40 weeks as compared to 15 weeks, respectively (8). However, additional work by Patchell et al. revealed that surgery alone, with no adjuvant radiation therapy, results in a local recurrence rate of nearly 50% (10).

While there have been improvements in stereotactic guidance, surgical techniques, as well as cross sectional imaging, the rate of recurrence for surgery alone for BrM is still close to 50% in more modern cohorts (11). To reduce this rate of recurrence, postoperative radiosurgery or radiotherapy is generally recommended, with recent trials informing practice and favoring SRS over WBRT because of the global neurocognitive deficits associated with WBRT (3, 12).

## Adjuvant radiosurgery and radiotherapy

WBRT as adjuvant therapy for surgically resected BrM has traditionally been considered the standard of care (8, 12). However, this type of therapy comes with the cost of substantial dose to functioning normal brain parenchyma leading to a decline in neurocognition. A recent cooperative randomized trial by Brown et al., N107c, revealed that postoperative SRS has equivalent OS in comparison to WBRT with median OS of 12.2 months vs. 11.6 months, respectively (*p* = 0.70). However, SRS was associated with statistically significant decreased rates of neurocognitive decline and functional independence (12). At 6 months post treatment, 85% of patients assigned to receive WBRT experienced cognitive deterioration as compared to 52% of patients assigned to receive SRS (*p* < 0.001). Postoperative SRS is thus now considered a standard of care and becoming a more frequently employed modality after surgically resected metastases.

However, WBRT has demonstrated improved local control in comparison to SRS (12). For example, in N107c, the 12 month surgical bed control rate was 61% in the SRS arm as compared to 81% in the WBRT arm (*p* < 0.001). Nevertheless, the rate of local recurrence with the combination of surgery followed by WBRT still approximates 20% at 12 months, including with 36 Gy in 12 fractions of adjuvant WBRT and in more modern series (8, 12). Furthermore, though treatment generally has improved with advancements in cross sectional imaging, surgical advances, targeted systemic therapy, and advanced delivery of radiation, the rate of recurrence after resection followed by SRS, as demonstrated by Mahajan et al. approximates 28% at 1 year and is even higher at 44% for lesions ≥3.0 cm (11). While the lower SRS doses utilized for this study may have been a factor in recurrence rates, local control for larger BrM has proven particularly challenging; and the potential local control advantage of WBRT previously described may not hold for larger metastases (12). Therefore, the potential for improvement in the treatment of BrM remains and alternative strategies are needed, with the goal to continue to improve local control while attempting to maintain the low toxicity profile associated with SRS.

## Alternative approaches, including neoadjuvant radiation (preoperative SRS)

While results of recent randomized trials have informed practice, the optimal approach to the management of surgically resected brain metastases has yet to be determined with centers providing multiple approaches to care, including observation after resection, adjuvant SRS, adjuvant WBRT, fractionated SRS, and neoadjuvant SRS, or alternative therapies besides or in addition to radiation therapy, such laser interstitial thermal therapy (LITT).

Fractionated SRS may offer advantages in comparison to a single fraction of radiosurgery including the potential to improve local control. Fractionation takes advantage of radiobiologic principles such as normal tissue repair and reoxygenation to deliver a higher dose of radiation with potentially similar or lower rates of toxicity including radiation necrosis, in comparison to single fraction SRS. The hypothesized improvement in local control with fractionated SRS could be anticipated to be similar to the differences in surgical bed control rates between WBRT and SRS described above. Retrospective series including an analysis by Minniti et al. have demonstrated improved 12 month local control of 91% in consecutive patients receiving fractionated SRS as opposed to a 12 month local control rate of 77% for patients receiving a single fraction (13, 14). Randomized comparisons are needed for further comparison.

A neoadjuvant (preoperative) approach to radiation therapy additionally offers the potential to improve rates of local control while decreasing rates of toxicity. Neoadjuvant radiation therapy is becoming popular across a number of disease sites. It is now a standard of care in sarcoma, rectal, esophageal, and pancreatic cancer (15–19). For example, the German rectal cancer study group reported results of a randomized trial which showed improved outcomes including better local control and less grade 3 or 4 toxicity with receipt of preoperative as compared to postoperative radiotherapy (16). It has also been shown that patients with resectable pancreatic tumors, who historically were not offered adjuvant radiation due to lack of proven benefit, do gain a significant benefit from a neoadjuvant approach (17). These findings are related to a number of advantages associated with neoadjuvant radiation therapy in comparison to adjuvant radiation therapy, and specific potential advantages in the preoperative vs. postoperative SRS setting for BrM are listed in Table 1 and explored in more detail below.

**Table 1:** Potential advantages and disadvantages of preoperative stereotactic radiosurgery compared to postoperative stereotactic radiosurgery.

**Potential advantages**	**Disadvantages**
↑ Local Control • Improved target delineation • Sterilization effect • Improved oxygenation ratio	Lack of Pathologic Confirmation Prior to SRS
↓ Leptomeningeal Disease • Sterilization effect	Not Compatible with Emergent Surgery (uncommon)
↓ Radiation Necrosis • Less normal brain irradiated • Resection of majority of irradiated tissue	↓ Wound Healing
↑ Systemic Control • Improved time to systemic therapy • Immunogenicity	

### Target delineation

**Figure 1:**
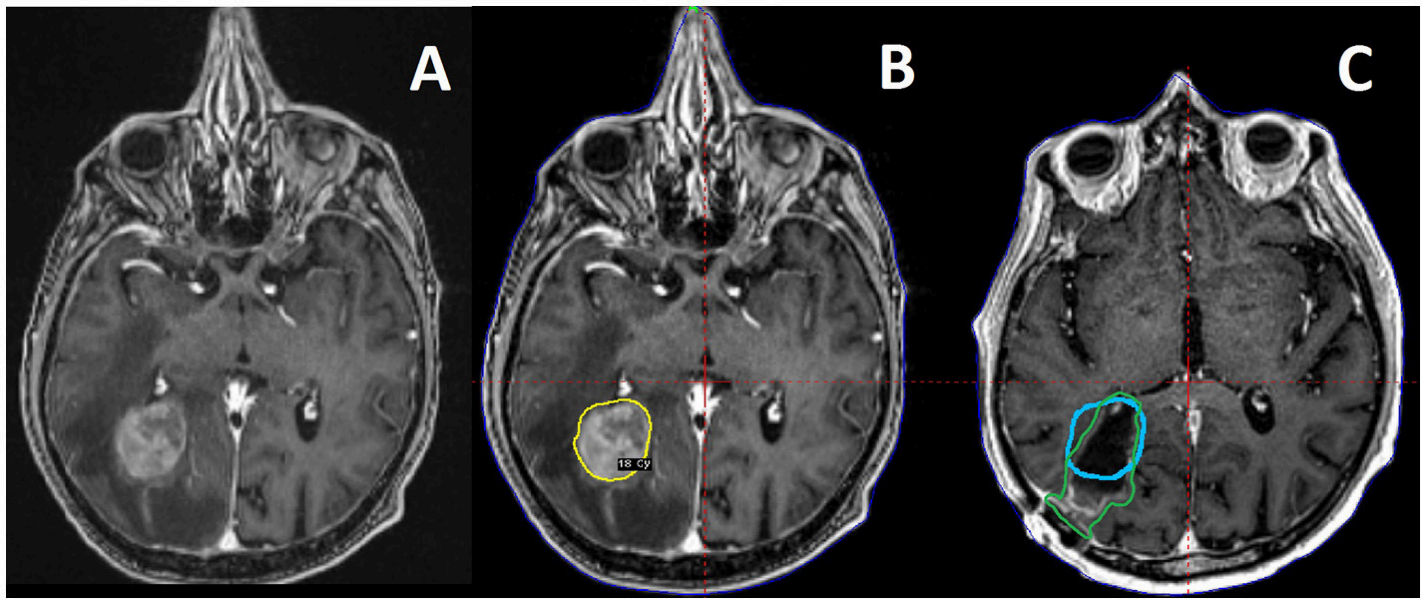
A 73 year old male patient with metastatic soft tissue sarcoma and three brain metastases, one large right occipital metastasis with associated edema **(A)**, as well as smaller tumors in the right motor strip and left temporal lobe (not pictured). He was treated with preoperative SRS **(B)** to 18 Gy to the 50% isodose line (20 Gy to the other, smaller tumors) followed by surgical resection of the occipital tumor the next day. Also pictured is a 3 month follow up MRI with the preoperative target depicted in blue, compared to the postoperative target depicted in green as per consensus guidelines **(C)**, demonstrating the change in the tumor cavity geometry after resection.

Preoperative SRS allows for less complex target delineation with less uncertainty when contouring an intact BrM. Postoperative SRS is more complex, attributable to the need to recreate a tumor bed, correctly interpret the altered appearance of manipulated dural surfaces in superficial cases, and decisions whether or not to include portions of the surgical tract for deeper lesions (Figure 1). Furthermore, the tumor bed can evolve postoperatively over time, adding the challenge of delineating residual tumor from postoperative changes, and contouring an irregularly shaped target whilst ensuring coverage of all areas of prior contact for previously resected BrM (20, 21). Retrospective analyses have found conflicting results in regards to the likelihood of surgical cavity increase vs. constriction post-operatively (22, 23). However, in either case, dynamic surgical bed changes after simulation but prior to the delivery of SRS represents an additional challenge in delineation unique to postoperative LINAC based SRS.

### Radiation necrosis

Radiation necrosis is a potential morbidity of SRS that can occur in up to 10–20% of patients and require further intervention, such as steroids, bevacizumab, resection, and/or LITT in select cases (24). The rate of radiation necrosis is proportional to radiation dose and the size of the lesion, with up to a 49.4% cumulative risk at 24 months of radiation necrosis (including asymptomatic treatment change) reported in lesions exceeding 1.0 cm treated with definitive SRS. Therefore, increasing prescription target dose to improve local control of BrM is limited by toxicity such as necrosis, particularly for larger lesions (24). Furthermore, newer systemic agents including immunotherapy and targeted biologics are being used more frequently and in combination with SRS. These agents generally have been shown to be efficacious, but in turn may increase the rate of radiation necrosis for patients receiving SRS (24, 25).

Preoperative SRS could reduce rates of necrosis relative to postoperative SRS (Table 2). After preoperative SRS, much of the irradiated rim of normal tissue receiving near target dose and surrounding the adjacent tumor will be resected at the time of surgery, potentially attenuating the availability of injured tissue and cytokine concentrations needed to catalyze radiation necrosis (Figure 1) (29, 30). In comparison, after surgical resection, postoperative SRS includes the surgical tumor bed and a rim or margin of normal tissue which receives prescription dose, potentially increasing the volume of normal brain irradiated and increasing risk of radiation necrosis (21).

**Table 2:** Studies Investigating Preoperative SRS.

**References**	**Patients**		**Outcomes**					
			**Local recurrence**		**Radiation necrosis**		**Leptomeningeal disease**	
			**Pre-op**	**Post-op**	**Pre-op**	**Post-op**	**Pre-op**	**Post-op**
Asher et al. (26)	*N* = 47		71.8%^~^	N/A	Considered Local Recurrence	N/A	0%	N/A
Patel et al. (27)	*N* = 66	*N* = 114	15.9%	12.6% (SRS)	3.09%	20.0% (SRS)	3.2%	8.3% (SRS)
Patel et al. (28)	*N* = 66	*N* = 36	24.5%^*^	25.1%^*^ (WBRT)	9.9%^*^	0%^*^ (WBRT)	3.5%	9.0%^*^ (WBRT)

*All timepoints are 1 year unless otherwise noted*.

* *Denotes 24 month timepoints*.

~ *Freedom from local recurrence*.

### Local control

As noted above, local recurrence following postoperative SRS remains high, especially for larger lesions, with a rate of 44% reported at 1 year for lesions of 3 cm or larger (11). Local recurrence is associated with worse OS in some series (12, 31). That local recurrence was decreased with postoperative WBRT in comparison to postoperative SRS (12) suggests viable cells can persist outside of the radiation treatment volume when delivery is performed in the postoperative setting, a problem that could be avoided with preoperative SRS. A circa 50% local recurrence rate following surgery alone (11) highlights the fact that tumor cells are frequently “spilled” at the time of surgery. When possible, en bloc resection technique can help mitigate that risk (32, 33). The unfortunate reality of many tumors, however, is that several factors from fragile cystic walls to dural contact, or vascular involvement, preclude effectively or safely performing an en bloc resection. Moreover, tumors approached via a trans-sulcal, trans-sylvian, interhemispheric, or transventricular approach present opportunities for dissemination of viable cells into the far reaches of those surgical access corridors. By operating after prior radiation, any tumor cells spilled are treated or “sterilized” and thus likely no longer replication competent, reducing the risk of recurrence beyond the treatment field.

Appropriate tumors in non-eloquent regions amenable to either en bloc resection, or generous resection of peri-tumor parenchyma, may be candidates for preoperative SRS dose escalation if rates of radiation necrosis risk could indeed be minimized through this approach. This would equate to improved control without increased toxicity.

Finally, the biologic effect of radiation therapy is in part dependent on generation of oxygen free radicals. As noted by Prabhu et al. oxygenation is decreased in the postoperative environment, making preoperative SRS theoretically more effective at comparable or even lower doses (34). The results of initial series of preoperative SRS are described in greater detail below. No local control benefit has been shown to date, however systematic, prospective evaluation is needed to reduce the impact of selection bias.

### Leptomeningeal disease

Leptomeningeal carcinomatosis can occur in the setting of BrM as well as other primary CNS malignancies. In the context of BrM, it is most common in certain malignancies such as breast cancer, particularly after neurosurgical resection, most especially in the posterior fossa (35). Development of diffuse leptomeningeal disease is associated with a particularly poor prognosis (36). WBRT has been associated with decreased rates of leptomeningeal disease in comparison to postoperative SRS. However, as described below, preoperative SRS appears to have similar rates of development of leptomeningeal disease compared to WBRT, without the associated neurocognitive deficit (28). This could be due to the above mentioned “sterilization” effect, which prevents the dissemination of replication-competent tumor cells at the time of surgery, potentially decreasing the rate of development of leptomeningeal disease in comparison to postoperative SRS.

### Logistics

The logistics of performing SRS in a recent postoperative patient may be complicated by competing needs to coordinate discharge or rehabilitation (rehab) placement needs with SRS, which is typically performed at the same institution, but on an outpatient basis. Rehab or skilled nursing facilities may be reluctant to accept a patient who has upcoming procedures scheduled. Moreover, patients in the postoperative setting may have pain control needs that put additional strain on both patient and staff. For facilities employing frame based SRS, frame placement may be easier and more comfortable without a tender or swollen incision, or underlying craniotomy, to negotiate. For these reasons, postoperative frame based SRS is rarely performed in the immediate postoperative setting, but usually 2–5 weeks after surgery, prolonging the total episode of care. An additional practical concern is the subset of patients undergoing resection who do not complete intended therapy with adjuvant SRS or WBRT, due to early progression or failure to follow up. Undergoing SRS 1–2 days prior to surgery has proven a logistically favorable and comfortable strategy for patients at our institution, allowing minimal time from diagnosis to completion of treatment.

Although pending SRS is not necessarily a contra-indication to chemotherapy, a prior series by Chang et al. comparing SRS to WBRT showed that WBRT resulted in an increased time to systemic therapy and may have been associated with worse overall outcomes as a result of this delay (37). As such, the impact of time to completion of management of BrM should be considered for potential impact on systemic management.

### Additional considerations—timing of therapy and immune activation

Radiation therapy is well-recognized to be both immunosuppressive and immunogenic (38). The precise timing, dose, fractionation, and ideal combination with systemic therapy to promote anti-tumor immune activation remain to be determined (39). The immune system has been shown to be important in controlling BrM, with immunotherapy, including dual checkpoint blockade, having demonstrated activity in the CNS (40). The combination of SRS and immunotherapy in the treatment of BrM is an area of active investigation. Ideally, the timing and dose of SRS in the treatment of BrM would consider optimizing potential benefits of radiation on any anti-tumor immune response. Thus, although preoperative SRS permits expeditious management, with surgery performed even as soon as the same day immediately following SRS, this–like postoperative SRS–may suboptimally exploit opportunities to harness the pro-immunogenic impact of tumor radiation (41).

This concept is well-illustrated in preclinical work by De La Maza et al. exploring radiation therapy prior to surgery and the short-term and long-term effect on the immune system. In a murine model, surgery alone or treatment with hypofractionated radiation therapy followed by surgery 1 day later resulted in zero tumor rejection to tumor re-challenge 90 days after treatment, demonstrating minimal long term immunologic memory response. However, hypofractionated radiation therapy followed by surgery 7 days later resulted in a 33% complete rejection of tumor on re-challenge 90 days after treatment, and this cohort of radiation therapy followed by surgery 7 days later had the lowest growth rate overall, as defined by tumor area, on tumor re-challenge. These findings were confirmed to be immunologic in origin, as they were markedly diminished when the mice were depleted of CD4+ T-cells (42).

How neoadjuvant radiation followed by surgery compares to surgery followed by adjuvant radiation therapy in terms of immune activation and so called *in situ* vaccination remains to be specifically tested. However, postoperative radiation therapy is likely less immunogenic compared to preoperative radiation therapy in the setting of recovery from a resection, including decreased neoantigen stimulation with likely only microscopic residual disease in the adjuvant setting (43). Finally, large ablative doses as prescribed in SRS may result in immune suppression in comparison to more moderate hypofractionation, making a fractionated pre-operative SRS approach with surgery 7 days or even more after radiation potentially the most immunogenic of all paradigms discussed (39, 44, 45).

Ultimately, the role and timing of achieving local control may require patient-specific considerations in the context of primary diagnosis, total intracranial and systemic disease burden, and immune status to optimize care. A patient's symptoms, performance status, treatment timeline, prior course, primary malignancy, type of systemic therapy and more may ultimately guide decision making, and where pre-operative SRS could be the ideal approach for a certain patient with BrM, another may benefit more so from a postoperative approach, including WBRT with memantine and potentially hippocampal avoidance in select circumstances (46, 47).

### Disadvantages

Preoperative SRS is not without limitations. One significant disadvantage of treatment of a lesion prior to resection is that the SRS is delivered before pathologic confirmation is available. This leads to the real possibility that a lesion which is radiologically and clinically consistent with a BrM is found not to be a metastasis, but a primary CNS malignancy, lymphoma, or an autoimmune condition, entities where the already delivered SRS would not be the treatment of choice for the diagnosis (and where radiation therapy may not be indicated).

Another disadvantage is that surgery is often indicated for highly symptomatic patients with mass effect, and preoperative SRS potentially delays the time from presentation to operation. This delay may be on the order of 6–48 h at centers with routine availability of SRS and neurosurgery and not significant for most patients. However, a preoperative SRS approach may not work for all patients nor be feasible in all care settings. Finally, preoperative SRS could lead to issues with wound healing compared to patients undergoing surgery prior to any therapy.

## Literature evaluating preoperative SRS and future trials

Limited data exist to evaluate the theoretical risks and benefits of preoperative SRS discussed above but generally support the safety, efficacy, as well as potential advantages of preoperative SRS. Asher et al. reported on 47 patients with many treated prospectively on trial, demonstrating the safety and efficacy of preoperative SRS, reporting local control rates of 85.6 and 71.8% at 12 and 24 months, respectively (26). Importantly, no perioperative morbidity or mortality attributable to the preoperative SRS was noted, although theoretical concerns (e.g., wound complications) are not infrequently raised. However, and again drawing on other existing literature from other disease sites, series have reported neoadjuvant radiation therapy was associated with decreased surgical complications and improved margin negative resection rates in certain instances (19, 48).

Preoperative SRS has been compared to postoperative SRS and postoperative WBRT in retrospective series. Patel et al. reviewed outcomes retrospectively of 66 patients treated with preoperative SRS in the largest series to date and in comparison to 114 patients who were treated postoperatively (27). In this analysis, preoperative SRS showed advantages in comparison to postoperative SRS. The preoperative cohort had statistically significant decreased rates of development of leptomeningeal disease (3.2 vs. 16.2% at 2 years), as well as statistically significant decreased rates of symptomatic radiation necrosis (4.9 vs. 16.4% at 2 years). However, this study showed no differences in local control between these two approaches (27). In a subsequent analysis, Patel et al. compared preoperative SRS to postoperative WBRT, finding no differences in local control or rate of development of leptomeningeal disease (28).

Further studies are necessary to compare preoperative vs. postoperative SRS. Phase II data described above support the safety and efficacy of pre-operative SRS, with mixed retrospective findings in regards to outcomes. When possible, future studies should continue to consider meaningful endpoints such as radiation necrosis, leptomeningeal disease, and local control, while taking into account considerations in timing, and utilizing correlative analysis to drive better understanding of the biologic response. Only with robust prospective and randomized data will we be able to determine if any of these hypothetical advantages to preoperative SRS are real and justify the known risks of preoperative treatment including SRS of a non BrM. If advantageous, eventual comparison will be needed to additional promising strategies such as fractionated SRS.

The NRG is currently developing a trial at the national level and several institutional trials are currently in development or enrolling, including Mayo Clinic MC167C, comparing preoperative SRS to postoperative SRS with a primary composite endpoint encompassing time to event of local recurrence, symptomatic radiation necrosis, or development of leptomeningeal disease.

## Conclusion

For surgically resected brain metastases, postoperative SRS has been adopted as the current standard of care in comparison to postoperative observation or WBRT. Recurrence rates after postoperative SRS, especially for larger BrM, are unfortunately high. Novel approaches including preoperative SRS may improve local control and decrease rates of leptomeningeal disease while also decreasing toxicity such as radiation necrosis. Further consideration regarding timing of intervention and prospective evaluation of preoperative SRS is warranted in prospective studies, which are currently underway.

## Author contributions

All authors listed have made a substantial, direct and intellectual contribution to the work, and approved it for publication.

### Conflict of interest statement

The authors declare that the research was conducted in the absence of any commercial or financial relationships that could be construed as a potential conflict of interest.
